# Positional plagiocephaly: results of the osteopathic treatment of 424 infants. An observational retrospective cohort study

**DOI:** 10.1186/s13052-024-01729-3

**Published:** 2024-09-07

**Authors:** Raffaella Panza, Filippo Piarulli, Valentina Rizzo, Federico Schettini, Maria Elisabetta Baldassarre, Antonio Di Lorenzo, Silvio Tafuri, Nicola Laforgia

**Affiliations:** 1 Department of Interdisciplinary Medicine - Section of Neonatology and Neonatal Intensive Care Unit, University of Bari, Bari, 70124 Italy; 2 Department of Interdisciplinary Medicine – Section of Hygiene, University of Bari, Bari, Italy

**Keywords:** Deformational plagiocephaly [Mesh], Plagiocephaly [Mesh], Infant [Mesh], Osteopathy, Medicine, osteopathic [Mesh]

## Abstract

**Background:**

Positional plagiocephaly is an asymmetrical flattened skull deformity whose incidence increased significantly in the last decades. Osteopathic treatment has been suggested to tackle early deformational sequences, in order to ensure the correct development of the child.

The aim of the study was to assess the effectiveness of osteopathic treatment of positional cranial deformities in infants.

**Methods:**

Retrospective observational study carried out at the Section of Neonatology and Neonatal Intensive Care Unit of the Department of Interdisciplinary Medicine of University of Bari, Italy in collaboration with a specialized pediatric osteopath.

**Results:**

424 infants were enrolled. Isolated positional plagiocephaly affected the vast majority of infants (n. 390, 91.98%); 34 patients (8.02%) were diagnosed with positional brachycephaly. Both infant groups (positional plagiocephaly and positional brachycephaly) had a median severity score of 3 (IQR: 3 – 3 and 2 – 3, respectively) and benefited from a median of 3 osteopathic sessions (IQR 3–4 and 2–4, respectively). Higher severity scores of positional asymmetries were significantly more common in preterm neonates (Pearson chi2: 11.58; *p*-value: 0.021) and in males (Pearson chi2: 10.06; *p*-value: 0.039).

**Conclusions:**

Significant improvements in positional cranial deformations of children were obtained after only five osteopathic treatments provided in the first months of life. The osteopathic treatment could positively impact the clinical history of patients with positional plagiocephaly and positional brachycephaly.

**Implication for practice:**

• Positional plagiocephaly is increasingly common among infants and may cause moderate to severe neurodevelopmental adverse effects.

• Osteopathic treatment may tackle early deformational sequences, in order to ensure the correct development of the child.

• Our study reveals that cranial asymmetry of infants with positional plagiocephaly is significantly reduced after only five osteopathic treatments provided in the first months of life.

• Osteopathic treatment should be offered as a first line approach to young infants diagnosed with positional plagiocephaly.

## Background

Plagiocephaly is an asymmetrical skull deformity mainly involving a unilateral flattening of the occiput. It can be synostotic (rare) or positional. The first, more serious, is due to premature closure of the cranial sutures; the second is characterized by changes in the cranial shape resulting from prenatal or postnatal mechanical forces. Posterior occipital plagiocephaly is prevalent today and has reached an incidence of 19.7% at 4 months. [1] Its greater diffusion is a direct consequence of the “Back to sleep” campaign launched by the American Academy of Pediatrics (AAP) in 1992 to reduce the incidence of sudden infant death syndrome (SIDS). [2] Risk factors for positional plagiocephaly (PP) include prematurity, prolonged labour, unusual birth position, assisted delivery, multiple birth, first-born child, neck problems, maternal age > 35 years, and male sex. [3]

**Fig. 1:**
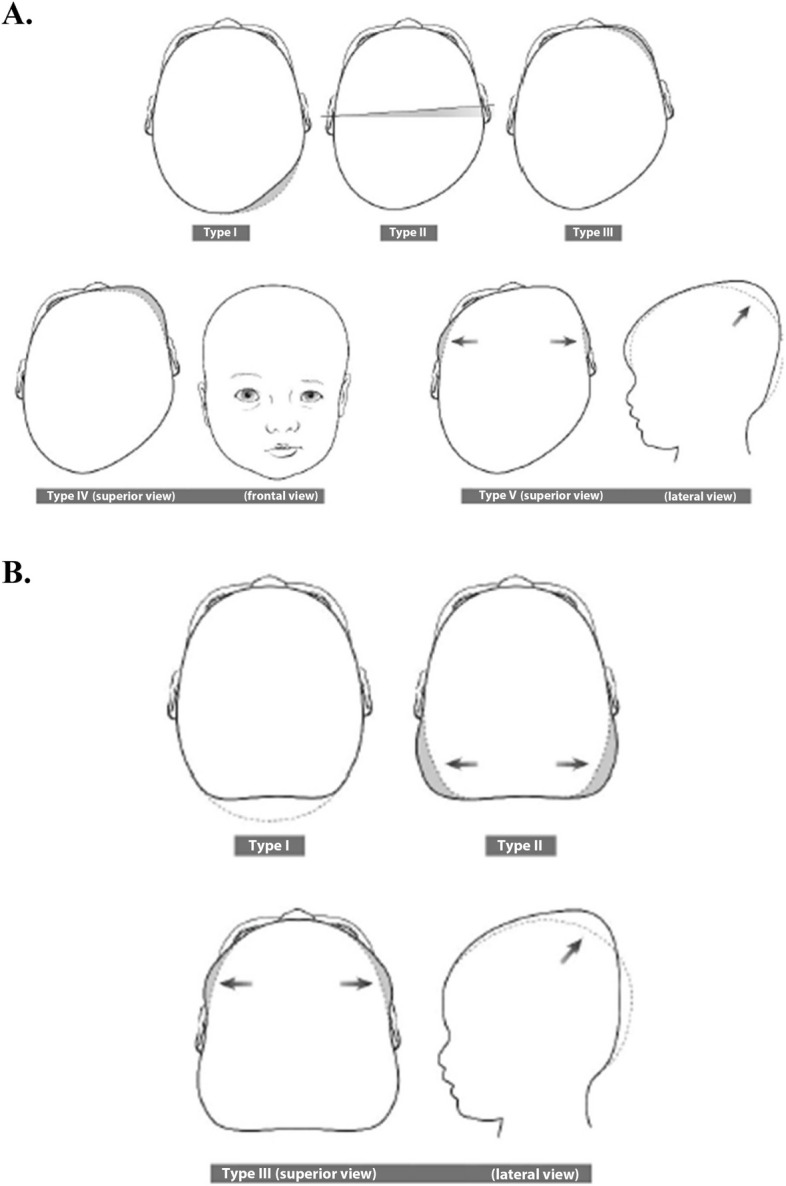
Argenta classification of deformational plagiocephaly (**A**) and brachycephaly (**B**) [7]

The classification of PP by Argenta is based on clinical observation alone and sorts deformational plagiocephaly on a scale from 1 to 5 (Fig. 1a). It evaluates the severity of the skull asymmetry, the position of the ear and the appearance of the face (ipsilaterally: protruding ear, wider eye, fuller lips, higher eyebrow). [4–6]

Occipital positional brachycephaly accounts for about 15% of cranial deformities in the neonatal period. It is classified separately on a scale from 1 to 3 (Fig. 1b), and predominantly affects large newborns or children whose motor activity is impaired by other causes (fracture of the leg or collarbone, neurological alterations or developmental anomalies). [8, 9]

The cranial asymmetry of plagiocephaly has an aesthetic impact but most importantly, if left untreated, can lead to altered neonatal development. Children with this condition are more prone to postural compensations, musculoskeletal dysfunction, visual disorders, temporomandibular joint defects, and neurocognitive delays. [10] However, to prompt an accurate diagnosis, physicians should always keep in mind that plagiocephaly could also be the consequence (not only the cause), of neurodevelopmental delays differentiating it in cases of craniosynostosis/congenital alterations of skull shape and genetic syndromes. [11–13] Among the conditions included in the differential diagnosis, congenital anomalies like neural tube defects have to be foremost considered. [14]

Without targeted intervention, most cases of plagiocephaly do not resolve on their own. [15, 16] Therefore diagnosis, prevention and treatment of positional plagiocephaly are of primary importance to ensure adequate neurodevelopment. The integrated multidisciplinary approach between neonatologists, osteopaths and, in the most serious cases, neurosurgeons must be timely, preferably around 3–4 months of life of the newborn, i.e. before the ossification of the synchondrosis at the base of the skull. [17–19]

In this retrospective observational study, we report the results of osteopathic treatment offered at a Neonatology and Neonatal Intensive Care Unit (NICU) in collaboration with a professional osteopath in the management of positional plagiocephaly.

The aim of the study was to assess the effectiveness of osteopathic treatment of positional plagiocephaly and its complications. We also evaluated the number of sessions necessary for the resolution of the craniofacial defect.

## Methods

This retrospective observational study was carried out at the Section of Neonatology and Neonatal Intensive Care Unit of the Department of Interdisciplinary Medicine of University of Bari, Italy. The study was conducted in collaboration with a specialized pediatric osteopath (F.P.).

We enrolled a cohort of premature and full-term infants diagnosed with plagiocephaly and related disorders in the period between January 2019 and December 2022.

Neonates with severe cranial and cerebral malformations (cranioschisis, cranial meningocele, encephalocele), birth trauma (cephalohematoma and birth tumor), severe genetically based craniosynostosis, trigonocephaly or metopic craniosynostosis were excluded.

Data were retrieved from medical records and entered into a Microsoft Excel file. All data sets were anonymised. Ethical approval was not necessary as this was an evaluation of current practice. Parental informed consent for data collection and picture publication was obtained. Statistical analysis was performed by Stata MP17.

### Osteopathic session

In plagiocephaly, distortion of the skull is often caused by membranous dysfunction affecting the reciprocal tension membrane system. Therefore, it is necessary to treat all skull components (membranes, joints and ligaments). In newborns or infants the fluid mechanics have not been excessively affected by the ongoing distortion. To release membranous tension, a variant of the venous sinus technique can be used. If this approach is adopted, the intervention is focused on the membranous structure in which the sinus was formed, rather than on the relationship between the sinus and the suture, or the fluid contained within the sinus. This is a direct approach aimed at balancing the membranous tensions between the mutual tension membrane components. This approach begins at the cranio-cervical hinge and proceeds until reaching the vault, resolving the membranous components first, then the bony ones and finally the fluid ones.

Any overlaps and protrusions of the sutures must be resolved. Suture dysfunction may be maintained by unresolved membranous tensions within the reciprocal tension membrane, or by external myofascial forces from the cranio-cervical hinge or the neck. The modeling technique described by Sutherland often does not prove effective until the influences mentioned above are eliminated. [20]

In most children with plagiocephaly, it is recommended to proceed with treatment of the neck and rib cage before moving on to the head. [21, 22]

### Descriptive statistics and normality assessment

Continuous quantitative variables were described as means (± standard deviation, SD). Discrete quantitative variables were described as medians (interquartile range, IQR). Categorical variables were described as percentages (proportion). The normality of continuous variables’ distribution was investigated via the skewness/kurtosis test. Following assessment, the distribution of the cohort’s children’s weight, length, cranial circumference and gestational age was found to be non-normal. All attempts at normalization were also unsuccessful.

Severity scores were described separately for each cranial abnormality. Since these scores are numerical, but act as severity categories, both proportions and median values were provided to describe them. However, due to the high number of repeated values, they were considered solely categorical variables for inferentiality’s sake.

### Inferential statistics

Confrontation among different groups were performed via the Chi-squared test, the Mann–Whitney test, or the Kruskal–Wallis test, according to the type of data involved. All inferential analyses were performed on the whole sample, without considering the difference in cranial abnormality. A two-tail *p*-value < 0.05 was considered indicative of statistical significance.

## Results

### Descriptive statistics

Four-hundred-thirty-four newborns with positional brachycephaly or plagiocephaly were enrolled in the study over the period between January 2019 and December 2022. Following exclusion of subjects with missing information regarding their severity score and/or expected number of osteopathic sessions, the remaining 424 subjects (males 257, 60.61%; median gestational age 39 (38 – 40) weeks; mean birth weight 3080.32 ± 655.51 g) were included in the statistical analysis. Neonatal demographics are shown in Table 1. Out of the 424 infants, 390 (91.98%) had positional plagiocephaly, and 34 patients (8.02%) were diagnosed with positional brachycephaly.

**Table 1:** Study population demographics

**Variable**		**Result**
Gender, n (%)	Male	257 (60.61)
	Female	167 (39.39)
Status at birth, n (%)	Preterm	55 (12.97)
	Term	369 (87.03)
Delivery mode, n (%)	Vaginal delivery	77 (18.16)
	Operative delivery	140 (33.02)
	C-section	207 (48.82)
Cranial abnormality, n (%)	Plagiocephaly	390 (91.98)
	Brachycephaly	34 (8.02)
Weight at birth (grams), mean (SD)		3080.32 (655.51)
Length at birth (centimeters), mean (SD)		48.98 (3.70)
Cranial circumference at birth (centimeters), mean (SD)		34.19 (2.73)
Apgar score, first minute of extra-utero life (pure number), median (IQR)		9 (8 – 9)
Apgar score, fifth minute of extra-utero life (pure number), median (IQR)		10 (10 – 10)
Gestational age at birth (weeks), median (IQR)		39 (38 – 40)
Osteopathic sessions (pure number), median (IQR)		3 (3 – 4)

**Fig. 2:**
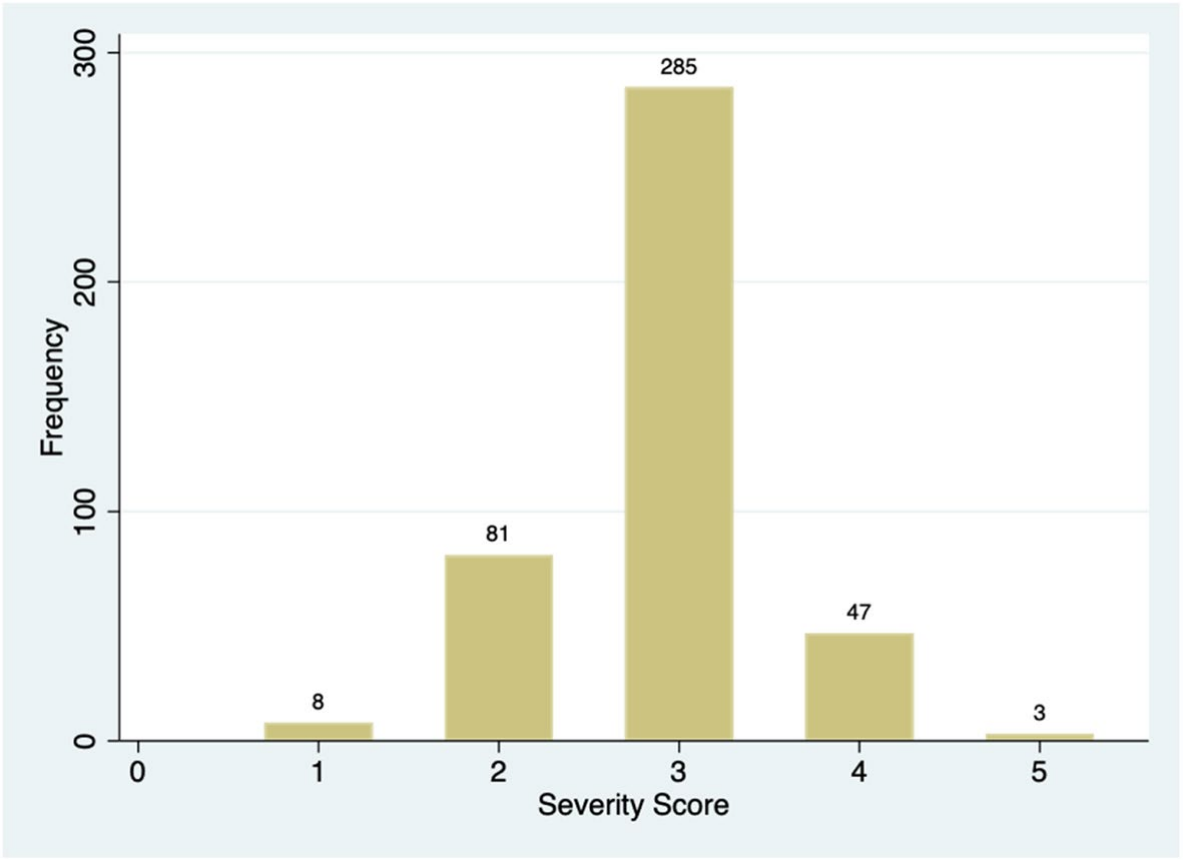
Distribution of severity scores across the population

**Fig. 3:**
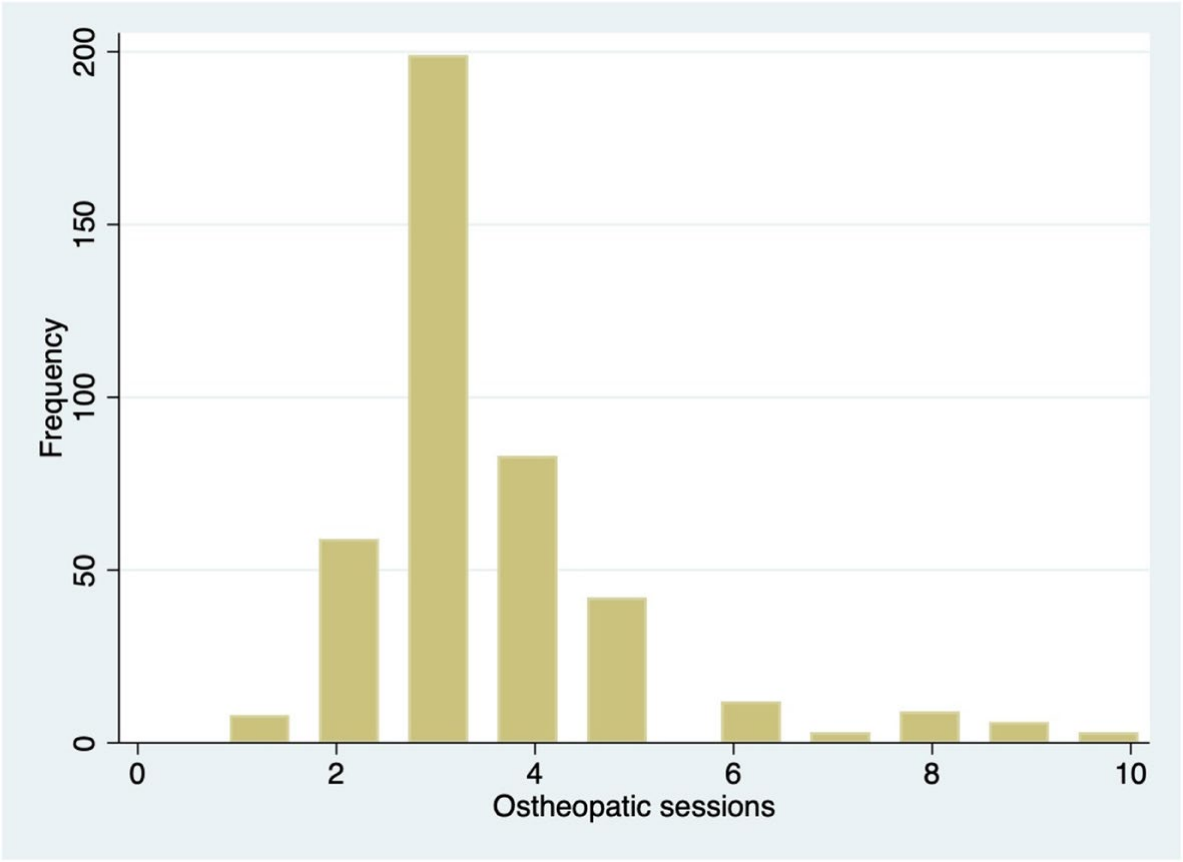
Distribution of received osteopathic sessions across the population

The distribution of severity scores in each subgroup of patients is described in Table 2. Distributions of severity scores and number of received osteopathic sessions across the population are also shown in graphic form in Fig. 2 and 3.

**Table 2:** Distribution of severity scores, by cranial abnormality

Cranial abnormality	Severity score	N	%
Plagiocephaly	1	6	1.54
	2	72	18.46
	3	265	67.95
	4	44	11.28
	5	3	0.77
Brachycephaly	1	2	5.88
	2	9	26.47
	3	20	58.82
	4	3	8.82

**Fig. 4:**
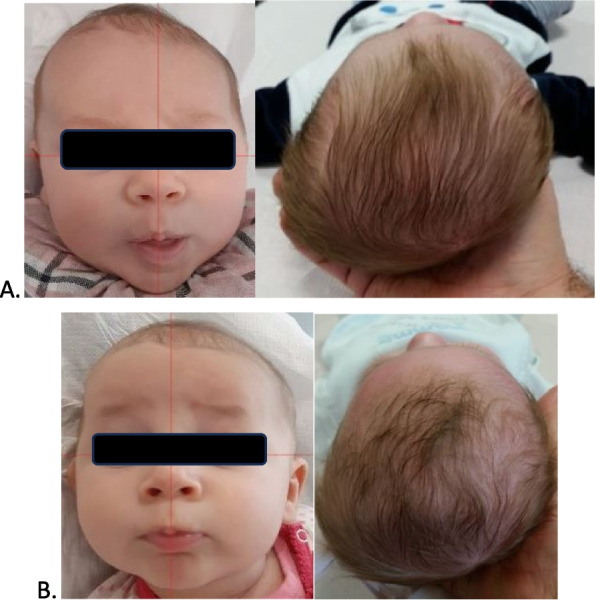
A patient with positional plagiocephaly before (**A**) and after (**B**) osteopathic treatment

Both infants with positional plagiocephaly and positional brachycephaly had a median severity score of 3 (IQR: 3 – 3 and 2 – 3, respectively). All patients improved after the osteopathic treatment with a complete resolution of the deformation. Patients with positional plagiocephaly or positional brachycephaly benefited from a median of 3 osteopathic sessions (IQR 3–4 and 2–4, respectively). An example of the results obtained is displayed in Fig. 4.

### Inferential statistics

The Chi-squared test highlighted a significant difference in the proportion of the various severity scores between term and preterm children (Pearson chi2: 11.58; *p*-value: 0.021), as well as between males and females (Pearson chi2: 10.06; *p*-value: 0.039). No significant difference was highlighted among different delivery modes (Pearson chi2: 9.72; *p*-value: 0.285). Results of Chi-squared tests are summarized in Table 3, 4 and 5.

**Table 3:** Distribution of severity scores, by status at birth

Severity score	Term		Preterm	
	***N***	***%***	***N***	***%***
1	8	21.68	0	-
2	73	19.78	8	14.54
3	251	68.02	34	61.82
4	34	9.21	13	23.64
5	3	0.81	0	-
Total	369		55

**Table 4:** Distribution of severity scores, by gender

Severity score	Male		Female	
	***N***	***%***	***N***	***%***
1	3	1.17	5	2.99
2	48	18.68	33	19.76
3	182	70.81	103	61.68
4	21	8.17	26	15.57
5	3	1.17	0	-
Total	257		167

**Table 5:** Distribution of severity scores, by delivery mode

Severity score	Vaginal delivery		Operative delivery		C-section	
	***N***	***%***	***N***	***%***	***N***	***%***
1	1	1.30	4	2.86	3	1.45
2	20	25.97	30	21.43	31	14.98
3	45	58.44	90	64.28	150	72.46
4	11	14.29	14	10.00	22	10.63
5	-	-	2	1.43	1	0.48
Total	77		140		207	

No significant differences were highlighted in terms of number of received osteopathic sessions in infants born via different delivery modes following assessment via Kruskal–Wallis test (*p*-value: 0.418). The Mann–Whitney test did not identify significant differences in the number of received osteopathic sessions between males and females (z: -0.61; *p*-value: 0.543) or term and preterm patients (z: -1.66; *p*-value: 0.097), either.

The mean number of received osteopathic sessions for each group is further detailed in Table 6.

**Table 6:** Mean received osteopathic sessions, by status at birth, gender and delivery mode

**Subgroup**	**Mean received sessions**	**Standard deviation ( ±**)	***P*-value**
***Delivery mode***			
Vaginal delivery	3.58	1.65	
Operative delivery	3.44	1.38	0.418
C-section	3.66	1.51	
***Gender***			
Male	3.56	1.50	0.543
Female	3.59	1.49	
***Status at birth***			
Preterm	4.05	1.98	0.097
Term	3.50	1.40	

Unsurprisingly, the Kruskal–Wallis test highlighted a significant difference in the number of received osteopathic sessions in subjects with different severity scores for their cranial abnormalities (*p*-value < 0.001). Further details are provided in Table 7.

**Table 7:** Mean received osteopathic sessions, by severity score

**Severity score**	**Mean received sessions**	**Standard deviation ( ±**)	***P*-value**
1	1.87	1.81	
2	2.76	1.04	
3	3.55	1.22	< 0.001
4	5.06	1.88	
5	8.00	2.65	

## Discussion

The incidence of positional plagiocephaly has been on the rise since the AAP rolled out the “Back to sleep” campaign for SIDS prevention. [2] Most forms of positional plagiocephaly can be prevented by following simple behavioral indications [23–25], aimed at avoiding the prolonged maintenance of some positions and guaranteeing a variability of pressure on the different regions of the skull. This information should be provided to parents before or during the neonatal period, when the child’s skull is more susceptible to deformation from external forces. On the one hand, parents must be informed about the importance of putting babies to sleep in the supine position to prevent the risk of SIDS, but at the same time it is important to spread the importance of “tummy time”, i.e. the proposition of the prone position when the newborn is awake. [26]

In cases of plagiocephaly or other craniofacial deformities that do not resolve with behavioral measures, pathological craniosynostosis/congenital alterations and genetic syndromes must be ruled out. Such pathological conditions may evolve even more severely to serious adverse short-and long term outcomes requiring neurosurgery, or leading to visual, hearing imapirments and/or life-threatening conditions, as occur for craniosynostosis or genetic syndromes with craniofacial anomalies or sequence malformations. In these cases diagnosis may be obtained with genetic as well as instrumental investigations (3DCT, in addition to sutures US). However, if the cranial abnormality is confirmed as “positional”, the osteopathic approach may represent a valid therapeutic option. [27–29] Osteopathic manual therapy allows to feel the physiological mobility at the level of the cranial sutures and the rhythm of the primary respiratory mechanism, defined as “craniosacral rhythm”. [22] This is an involuntary body rhythm, automatic and independent of respiratory and cardiac rhythms, which allows the osteopath to treat the body as a functional unit. Craniosacral motion is transmitted directly from the sphenobasilar synchondrosis to other cranial bones via cerebrospinal fluid and intracranial meningeal membranes. The osteopath, placing each of his fingers on the nearby bones, perceives the mobility between each bone segment; in this way he also recognizes any tissue shortenings (fascia and neck muscles) that accompany postural deformation. Some key elements of osteopathic treatment for positional plagiocephaly or positional brachycephaly include: normalization of the skull base; optimization of vertebral alignment and normal head/neck mobility (without resorting to the thrust technique); normalization of cranial membranes, cranial sutures and intraosseous lesions. [30] The formation of skull bones begins with tiny centers of ossification scattered throughout a matrix of connective tissue. The sutures between the skull bones are plastic and flexible, so that the bones can overlap during the remodeling process. [31] The goal of osteopathic treatment is to remove any impediments to these mechanisms and facilitate the homeostatic processes of the body. [32] Osteopaths diagnose and treat positional asymmetries, such as plagiocephaly or brachycephaly, by identifying and treating joint motion limitations with the aim of optimizing symmetry in the growing child. The timeliness with which treatment is carried out in infants and newborns affected by positional cranial deformities appears to influence the outcome. If tissue dysfunctions can be resolved and tensions can be balanced before certain growth phases, the body can restore tissue balance in the distorted area. Conversely, plagiocephaly, if not treated, negatively affects the cervical spine, trunk and postural strategies during the child’s growth, making it much more difficult for the dysfunctions to resolve.

The present study demonstrates that osteopathic treatment is successful for neonates and infants with positional cranial deformities. Higher severity scores of positional asymmetries are significantly more common in preterm neonates (Pearson chi2: 11.58; *p*-value: 0.021) and in males (Pearson chi2: 10.06; *p*-value: 0.039). However, the vast majority of patients with either positional plagiocephaly or positional brachycephaly recovers after less than 5 osteopathic treatment sessions. The total number of osteopathic sessions required to improve the positional asymmetry is significantly and directly associated with the severity score, whereas no significant associations were found with either gender or prematurity. Similarly, a recent retrospective study found that a series of osteopathic treatments determines a significant reduction of skull asymmetry and occipital flattening, as indicated by a decrease of the cranial vault asymmetry index from 6.809 (± 3.335) (Grade 3 severity) at baseline to 3.834 (± 2.842) (Grade 2 severity) after treatment. [33]

The success of this approach, in accordance with the short therapeutic timing and the absolute non-invasiveness of the techniques, makes osteopathy the gold standard in the treatment of nonsynostotic positional plagiocephaly or brachycephaly. The benefits obtained from osteopathy are not only related to the esthetical imperfections that positional plagiocephaly or positional brachycephaly can cause, but also and above all to the functional disorders associated with it. Early assessment and diagnosis of positional deformation are essential to prevent significant delays in gross motor development (e.g. sitting up, rolling back to side, crawling), neck muscle dysfunction, and decreased muscular tone. [34–36] An association between PP and mandibular asymmetry has been reported, possibly secondary to the rotation of the cranial base and anterior displacement of the temporomandibular joint. [37] Therapeutic decisions and managements may vary according to the severity of PP and the timing of treatment. Nonetheless, earlier interventions are usually more effective. [15, 16, 38] A pilot study revealed a 50% mean reduction in asymmetry in 12 infants after four osteopathic sessions (60 min each) scheduled 15 days apart (± 4 days). [39] To date, most studies support osteopathy effectiveness in the neonatal and paediatric population, however the small sample sizes limit the soundness and reproducibility of findings. [39–43]

## Conclusions

The present study demonstrates significant improvements in cranial asymmetry of children with PP after only five osteopathic treatments provided in the first months of life. Osteopathy should be reserved on the one hand for early deformational sequences and on the other for the failure of preventive measures, in order to ensure the correct morphological and functional development of the child. Our study highlights the importance of the osteopathic approach in the treatment of positional plagiocephaly in infants and in preventing the risk of the onset of multifunctional complications resulting from it. It is important to monitor the asymmetries of the face and the motor functions of the infant in order to make an early clinical-neonatological diagnosis of plagiocephaly and plan its management. Cranial deformities are in fact related to a greater incidence of gastrointestinal disturbances, alterations of visual function, idiopathic scoliosis and more severe complications, such as cognitive and psycho-motor development deficits. Therefore, although positional plagiocephaly in itself is not a serious pathological condition, its possible long-term esthetic and functional repercussions should not be underestimated. Further collaborative studies are needed to create a standardized osteopathic treatment protocol which could positively impact the clinical history of patients with plagiocephaly and other craniofacial deformities.

## Data Availability

The datasets generated and analysed during the current study are available from the corresponding author on reasonable request.

## Abbreviations

AAP: American Academy of Pediatrics
SD: Standard deviation
SIDS: Sudden infant death syndrome
IQR: Interquartile range
NICU: Neonatal Intensive Care Unit
PP: Positional plagiocephaly
